# The influence of social context on the treatment outcomes of complementary and alternative medicine: the case of acupuncture and herbal medicine in Japan and the U.S.

**DOI:** 10.1186/s12992-015-0103-2

**Published:** 2015-04-25

**Authors:** Jae-Mahn Shim

**Affiliations:** Department of Sociology, University of Seoul, 163 Seoulsiripdae-ro, Dongdaemun-gu, Seoul 130-743 Korea

**Keywords:** Complementary and Alternative Medicine (CAM), Japan, U.S, Different Treatment Outcomes of CAM, Institutionalization

## Abstract

**Background:**

Complementary and alternative medicine (CAM), such as acupuncture and herbal medicine, is popular in many countries. Yet, treatment outcomes of CAM are found to vary significantly between medical trials in different social environments. This paper addresses how the social organization of medicine affects medical treatment outcomes. In particular, it examines the extent to which two popular complementary and alternative medicine (CAM) interventions (acupuncture and herbal medicine) are coordinated with biomedicine and how coordination characteristics are related to the treatment outcomes of the two CAM interventions.

**Methods:**

This paper conducts an archival analysis of the institutional settings of the CAM interventions in Japan and the U.S. It also conducts a systematic content analysis of the treatment outcomes in 246 acupuncture reports and 528 herbal medicine reports that are conducted in Japan or the U.S. and registered in the Cochrane Library’s Central Register of Controlled Trials (CENTRAL), and 716 acupuncture reports and 3,485 herbal medicine reports that are from Japan or the U.S. and listed in MEDLINE. It examines the association between the treatment outcomes of the two interventions and the geographical location of the reports; it also explores how the institutional settings of the interventions are related to the treatment outcomes.

**Results:**

Japanese herbal medicine is integrated into the national medical system the most and American herbal medicine the least; American acupuncture and Japanese acupuncture fall in the middle. Treatment outcomes are the most favorable for Japanese herbal medicine and the least favorable for American herbal medicine. The outcomes of American acupuncture and Japanese acupuncture fall in the middle.

**Conclusions:**

The co-utilization of CAM with biomedicine can produce difficulties due to tensions between CAM and biomedicine. These difficulties and subsequent CAM treatment outcomes vary, depending on how CAM is institutionalized in relation to biomedicine in the national medical system. Coordinated CAM interventions are more likely to be effective and synergic with biomedicine, when compared to uncoordinated ones.

## Background

Complementary and alternative medicine (CAM), such as acupuncture and herbal medicine, is popular in many countries [1-3]. Health care systems have accordingly organized CAM along with mainstream biomedicine in various ways [4-8]. Treatment outcomes of CAM are found to vary significantly between medical trials in different social environments [9-11]. However, social scientific interest in the effectiveness and reliability of CAM as a medical resource [12-16] has paid little attention to an organizational sociological insight that the social organization of medicine can affect the outcomes of medical treatments [17,18]. I draw on this organizational sociological perspective and investigate how variations in the effectiveness of CAM are related to differences in the institutionalization of CAM into a national health care system.

This empirical inquiry is situated at the intersection of two traditions in medical sociology. One, or the sociology of medicine, is relatively established in the discipline with its tenet being the social construction of medicine [19-22]. Its insight is consistent with the observation that CAM has been constructed and institutionalized differently into health care systems in the world. The other, or the sociology of the effects of medicine, is relatively nascent in the literature. Early studies have only demonstrated the significance of social factors intervening in the outcomes of biomedicine, by proposing cultural accounts of the successes or failures of biomedical treatments [23-26], bio-social models of treatment outcomes [27], and the society–gene interaction model of genetic causation [28]. It has yet to be examined how social contextual influences exist among the puzzlingly varying treatment outcomes of CAM.

At the conjuncture of the two traditions, I hypothesize that the degree to which CAM is institutionalized within the mainstream biomedical health care system has consequences for the effects of CAM. This hypothesis is based on existing ethnographic observations that the institutional settings of CAM affect how users perceive and what they expect from CAM treatments [29] and that negative social labeling of illnesses and unconventional medicines may lead to secretive and uninformed uses of informal medical resources ([30]: 1814). Most importantly, this hypothesis pays heed to the theoretical and political tensions and conflicts between CAM and biomedicine in addressing illnesses [20,31-33]. In addition, the tensions and conflicts can vary, depending on the extent to which CAM is institutionalized within the dominant biomedical system. When institutionalized, CAM is likely to have less tensions and conflicts with biomedicine. Varying tensions and conflicts can subsequently shape how CAM is practiced alongside biomedicine in specific treatment contexts, which has in turn consequences for the treatment outcomes of CAM.

The purpose of this paper, therefore, is twofold. First, I investigate quantitatively whether there is a discernible correspondence between CAM institutionalization and CAM treatment outcomes. Second, I provide a sociological explanation of the correspondence by qualitatively revealing specific treatment episodes from reports in medical journal databases.

I take a methodological cue from the medical science community (i.e. the meta-analytic approach based on a systematic content analysis) and adapt it into a sociologically informed meta-analysis. The medical science community has responded to the inconsistent outcomes of medical interventions with meta-analyses of research findings [34-36]. Meta-analyses are expected to produce generalizable evidence by adjusting inconsistent outcomes to differences in trial design (e.g. the size of trial subjects), outcome measures, or the statistical models used. Indeed, some early agreements have ensued with regard to the efficacy and safety of CAM interventions [37-39].

Medical meta-analyses, however, have two significant limitations compared to medical sociological interests. First, they neglect the treatment outcomes reported by users and practitioners in real-world medical practices outside “scientific” clinical trials. Aiming to re-calibrate the effects of only technological interventions arguably independent of social circumstances, medical meta-analyses focus on clinical trials (e.g. randomized controlled trials [RCTs]) where the impacts of the social environment are designed to be non-existent. Thus, they have excluded treatment outcomes that are reflected in patient case reports and practitioner commentaries in medical journals. Treatment outcomes in real-world treatment settings outside clinical trials and those from practical medicine in clinics rather than theoretical science in laboratories have been obscured.

Second, the variations in treatment outcomes across doctors’ offices and hospitals have also been obscured. Medical meta-analyses aim at generating consensus rather than identifying disagreements. The limitation of this approach to re-calibrating the universal – rather than differential – effects of medical interventions is evident, when acupuncture and herbal medicine of an identical quality and of an identical trial design (a randomized controlled trial [RCT]) are intriguingly found to produce varying and even conflicting treatment outcomes at different trial sites [10,11].

Through a sociological meta-analysis, I purport to systematically document the varying outcomes of CAM in two different social contexts. My analysis aims not to bracket these variations out of consideration, as medical meta-analyses have done in search of the universal effects of CAM interventions. Instead, I examine two popular CAM treatments, acupuncture and herbal medicine, to show how divergent their treatment outcomes are between Japan and the U.S. I then examine how these treatment outcomes are related to the institutional environment of the interventions in the two countries. I stress the significance of coordinating acupuncture and herbal medicine treatments with mainstream biomedicine in order for the CAM interventions to produce tangible health care benefits. I also argue for the relevance of a sociological meta-analysis to identify and explain the differential outcomes of medicine.

### Data and methods

#### Case selection: acupuncture and herbal medicine in Japan and the U.S.

I examine the treatment outcomes of acupuncture and herbal medicine in Japan and the U.S. for two reasons. First, acupuncture and herbal medicine are institutionalized differently in the two countries. Theoretically, acupuncture and herbal medicine together form the whole system of “oriental medicine” or “traditional Chinese medicine”. Japan and the U.S. have, however, institutionalized acupuncture and herbal medicine being disconnected from each other. Furthermore, the two countries show reversed institutional prominence between acupuncture and herbal medicine. Acupuncture has been incorporated into the American medical system as one of the legitimate medical practices, whereas herbal medicine still remains illegitimate. In Japan, to the contrary, both have been institutionalized into the national medical system, where herbal medicine has gained the status of a legitimate medicine as *kampo* whereas acupuncture has been institutionalized as a second-class pseudo-medicine.

Second, studies suggest cross-national differences between East-Asian countries and Euro-American countries in the degree to which acupuncture and herbal medicine are found to be effective [9,40,41]. Among these countries, Japan and the U.S. are two of the countries that conduct most medical research in the world [42]. Thus, they do not only provide the richest empirical reports about the treatment experiences of acupuncture and herbal medicine but also constitute an interesting pair that invites a sociological investigation of how different institutional settings are related to treatment outcomes.

#### Meta-analysis

To specify the treatment outcomes of acupuncture and herbal medicine in the two countries, I have used medical journal papers as the unit of analysis. Medical journal papers are from two prominent medical databases: the Cochrane Library’s Central Register of Controlled Trials (CENTRAL) (http://www.thecochranelibrary.com/) and the U.S. National Library of Medicine’s MEDLINE (http://www.nlm.nih.gov/bsd/pmresources.html) both of which hold medical reports from across the world.

As of May 2012, there are 673,964 controlled clinical trials registered in CENTRAL. I have retrieved 5,243 trials of acupuncture, which refer to “acupuncture” in the title, abstract, or keywords. Of the total acupuncture trials, 108 and 138 trials have been conducted by researchers based in Japan and the U.S., respectively. I have retrieved a total of 5,812 trials on herbal medicine, which refer to “herbal medicine”, “herbal therapy”, “herbal supplement”, “herbal preparation”, “herbal remedy”, “kampo”, “kanpo”, “medicinal plant”, or “plant extract” in the title, abstract, or keywords. Of these trials, 228 have been conducted by researchers in Japan and 300 in the U.S. I analyze the content of all 246 acupuncture trials and 528 herbal medicine trials. I focus on four measures: treatment effectiveness, biomedicine–CAM synergy, adverse treatment outcomes, and negative biomedicine–CAM interactions.

As of 2012, the second database, MEDLINE, includes over 700,000 records in the subset of CAM (“Complementary Medicine”) that is developed by the U.S. National Center for Complementary and Alternative Medicine (NCCAM) (for the detailed strategies used for the subset, see http://www.nlm.nih.gov/bsd/pubmed_subsets/comp_med_strategy.html). Unlike CENTRAL which lists only controlled clinical trials, MEDLINE covers various reports, such as patient case reports, practitioner opinions and comments, news reports, policy analyses, and historical reviews (see Table 1 for details). Thus, MEDLINE uniquely incorporates reports from users and practitioners in real-world medical practices outside of controlled laboratorial trials. Based on Boolean search queries where I utilize the MeSH (Medical Subject Headings) terms for CAM modality and geographical location (see Appendix for each query in detail), I have retrieved 115 reports of acupuncture practiced in Japan and 601 reports in the U.S. as of May, 2012; there are 1,071 reports of herbal medicine from Japan and 2,414 reports from the U.S. Table 1 presents how these reports are distributed across different report types.

**Table 1:** **Types of the MEDLINE Reports Included in the Analysis**

**Report Type**	**Reports about Acupuncture**		**Reports about Herbal Medicine**	
	**from Japan**	**from the U.S.**	**from Japan**	**from the U.S.**
Clinical Trials	10 (8.3%)	27 (4.4%)	42 (3.9%)	48 (2.0%)
Case Reports	5 (4.2%)	13 (2.1%)	12 (1.1%)	54 (2.2%)
Professional Opinions	3 (2.5%)	45 (7.3%)	31 (2.9%)	176 (7.2%)
Reviews	13 (10.8%)	110 (17.8%)	58 (5.4%)	331 (13.5%)
Historical Articles	33 (27.5%)	39 (6.3%)	97 (8.9%)	178 (7.2%)
News Articles	0 (0.0%)	24 (3.9%)	6 (0.6%)	194 (7.9%)
Unclassified	51 (42.5%)	343 (57.1%)	825 (77.0%)	1433 (59.4%)
Total	115 (100.0%)	601 (100.0%)	1071 (100.0%)	2414 (100.0%)

Note: Report types are based on the categories in the “Article Type” filter in MEDLINE (accessible online at PubMed). The MEDLINE classification system has dozens of categories that are not unidimensional or mutually exclusive. Some of them are relevant to considering the medical scientific quality of the reports (e.g. report types included in this table), while others are not (e.g. funding sources of the reports). I include controlled clinical trials, randomized controlled trials, and clinical trials under the type of “clinical trials;” “case reports,” historical articles,” and “news articles” are distinct categories of themselves as classified in the system; “professional opinions” include comments, letters, editorials, interviews, and addresses.” “Reviews” include unsystematic reviews, systematic reviews, and meta-analyses. When a report is not indexed with any of these categories, it is grouped into “unclassified.”

I analyze the content of all 716 acupuncture reports and 3,485 herbal medicine reports in the same four measures. The four measures have been operationalized in the following. I apply the method of “closed coding” [43] in which I code each report 1 or 0 for each of the four measures, depending on whether the report provides a positive or a negative answer to a pre-determined question for the measure.

First, *treatment effectiveness* measures whether acupuncture or herbal medicine intervention was found to be effective in treating or preventing medical conditions, compared to the outcomes in pre-treatment or control groups. When a paper found the intervention effective, the paper is coded 1 (0 otherwise).

Second, *biomedicine–CAM synergy* refers to the presence of positive outcomes from the concurrent use of CAM with biomedicine treatments (coded 1; 0 otherwise). From the data, two types of synergy have emerged. First, the combined treatments of biomedicine and CAM were often found to be effective when the lone treatment of either biomedicine or CAM was not. In other cases, the effectiveness of combined treatments was found greater than that of either lone treatment. Second, synergy was shown in cases where CAM was used to relieve the side effects of biomedical treatments and to help patients to continue their biomedical treatments. Without CAM and its synergic effects, it would have been difficult to continue the biomedical treatments.

Third, *adverse treatment outcomes* refer to the presence of unforeseen adverse medical events from CAM (coded 1; 0 otherwise). I code adverse events as were reported in clinical trials in CENTRAL; for reports from MEDLINE, I use the MeSH term “adverse effects” to code the presence.

Fourth, *negative biomedicine–CAM interactions* measure the presence of adverse treatment interactions between biomedicine and CAM (coded 1; 0 otherwise). Whereas adverse treatment outcomes refer to adverse events from CAM only, this measure reflects adverse events that occur through the interactions between biomedicine and CAM. I code the interactions as reported in CENTRAL trials; I use the MeSH term “herb–drug interactions” for MEDLINE reports. Negative herb–drug interactions are defined as adverse outcomes that are not expected of separate applications of each of the concurrently used drug and herbal medicine ([44]: 631).

Through this coding process, each report is given a value of 1 or 0 for each of the four measures of treatment outcomes. In addition, each report is coded whether it is about acupuncture or herbal medicine (*modality*); whether it is from Japan or the U.S. (*geographical location*). Then, I use Pearson’s χ^2^ test of independence for the relationship between the geographical location (Japan vs. the U.S.) and each of the four measures of treatment outcomes and that between modality (acupuncture vs. herbal medicine) and each of the four measures. This test gives quantitative answers to whether there are cross-national differences in CAM treatment outcomes between Japan and the U.S. and how they correspond to differences in the institutional settings of acupuncture and herbal medicine. This quantitative test of independence between the institutional settings of CAM treatments in different countries on the one hand and the treatment outcomes on the other constitutes the first unique feature of the sociologically informed meta-analysis that this paper performs.

In addition, I complement this quantitative analysis with a qualitative analysis, by extracting the details of institutional settings from the reports. I apply the method of “open coding” [43] to discover themes related to the treatment contexts in which acupuncture or herbal medicine is practiced. For each report, therefore, these contexts are coded descriptively in text. Several themes have emerged from this open coding process that explain the results from the quantitative analysis, such as practitioner skills, the holistic practices of CAM, the organizational coordination of CAM with biomedicine, patient–physician communication of CAM use, and cross-cultural utilization of healthcare services. This open coding process, which focuses on the treatment contexts and subsequently extracts these themes emerging from the contexts, is the second unique feature of the sociologically informed meta-analysis.

The emergent themes from the open coding process are all grounded in the reports that I refer to with unique document identifiers: CENTRAL-generated CNIDs (i.e. 8-digit numbers led by “CN–”) and MEDLINE-generated PMIDs (i.e. 8-digit numbers led by “PMID”). With these unique identifiers, one can easily access the full reports in each of the online databases (use CNIDs in the search box at http://www.thecochranelibrary.com/view/0/index.html; use PMIDs in the search box at http://www.ncbi.nlm.nih.gov/pubmed).

### Results from the comparison of the institutional settings in Japan and the U.S.

#### Japan: acupuncture and herbal medicine integrated into the medical system

Japanese herbal medicine *kampo* literally means “Chinese or Oriental (*kam*) theory or therapeutics (*po*)” that does not only include herbal medicine but also acupuncture, cupping, and moxibustion. Since the 1970s, however, *kampo* refers in practice to herbal medicine only. Herbal medicine has since managed to remain at the center of traditional oriental medicine whereas other elements, such as acupuncture and moxibustion, have been marginalized.

From a historical perspective, however, it was herbal medicine, rather than acupuncture, that was first marginalized. First established as traditional medical practices in the 6th century [45], acupuncture and herbal medicine underwent major tumults during the Meiji government-driven medical modernization (1868–1912). Meiji legalized only medical practices that were adopted from the Netherlands and Germany. Both acupuncture and herbal medicine were denied legal recognition.

Around 1885, however, acupuncture gained official recognition from prefecture governments as a vocational course for the blind and not as a legitimate medical tradition. The reinstatement of acupuncture in the vocational context culminated in the 1911 legislation that permitted the practices of acupuncture and moxibustion as business entities and not as medical practices. It was under the post-World War II American rule that acupuncture and moxibustion were recognized as medical practices that required formal education and licensure ([46]: 8–9).

Acupuncture education was further formalized, as it expanded into universities like *Meiji Shinkyu Daigaku* established in 1983. Subsequently, the Japanese government initiated a nation-wide standardized licensure in 1993, replacing the fragmented systems overseen by local governments. Acupuncture has since maintained the institutional recognition. Currently, there are 80 three-year vocational schools, 6 four-year colleges occasionally with post-graduate programs, and medical schools all teaching acupuncture as a required or elective course [47-49]. It is estimated that there are about 130,000 licensed and 80,000 practicing acupuncturists in about 50,000 clinics throughout Japan [50]. National surveys estimate that 6–7% of Japanese adults visit acupuncture clinics [51,50].

To the contrary, herbal medicine remained outlawed for a century from 1868 to 1967. Then, four traditional herbal medicine (*kampo*) formulae in 1967 and 43 formulae in 1976 were suddenly recognized by the national government and sanctioned in the national insurance system, comparable to pharmaceutical drugs [52,53]. Several accounts exist for this sudden institutional acceptance of *kampo* after a century of long neglect, some of which stresses the strong lobbying of the Japanese Medical Association [54], the needs of patient groups, or the business interests of *kampo* manufacturers [53]. *Kampo* has since been prescribed only by medical doctors like biomedical drugs.

This reinstatement of herbal medicine into the center of medicine has in fact been accompanied by several deliberate – although hidden – efforts. First, doctors at Meiji-era medical schools kept studying *kampo* and conducting research with students, even though they could not teach it officially ([55]: 7). The successful pharmacologic isolation of alkaloid ephedrine from the *kampo* herb *mao* (Ephedra sinica) in 1887 was another achievement ([56]: 52). Facing denial from the Meiji government, *kampo* researchers appropriated government-espoused pharmaceutical models for underground research into *kampo* and produced the achievement. Second, unofficial *kampo* research by three distinct groups of medical doctors brought about a historic event in *kampo,* or the publication of *The Practice of Clinical Kampo Medicine* in 1941 ([55]: 7–8). The publication provided a list of formulated *kampo* prescriptions that are matched to patient symptoms. Thus, physicians without theoretical knowledge of *kampo* were able to practice it with a ready-made set of symptom-specific formulae. This publication was followed by the Japanese Medical Association’s endorsement of *kampo* for its acceptance into the national insurance system.

Several regulatory measures, comparable to those of biomedical drugs, further promoted the full institutionalization of herbal medicine, such as the introduction of toxicity tests, three-phased clinical trials, and efficacy evaluations [53,57]. Currently, 148 formulae and over 200 herbs are being supported by the national health insurance system [52]. Herbal remedies can be purchased from pharmacies with the support of national insurance when they are prescribed by a physician or without the insurance support when there is no physician prescription. About 70% of physicians reportedly prescribe *kampo* (*Nikkei Medical* October 2003: 33–39). The total annual expenditure for *kampo* is estimated to be more than one billion dollars [56]. Following a government mandate, 79 out of the 80 Japanese medical schools teach *kampo* courses. There are 2,420 *kampoi* (or, board-certified physicians for *kampo* practices) and about 6,000 non-certified *kampo*-practicing physicians among the total number of 280,000 physicians (http://www.jsom.or.jp/).

#### The U.S.: acupuncture integrated in the system while herbal medicine being an outcast

Not until the 1970s did acupuncture and Oriental medicine (AOM) begin to be officially recognized by state governments. AOM had remained as unofficial and underground medical practices known only to Asian immigrant communities [58]. From the 1970s, acupuncture was readily decoupled from the whole system of Oriental medicine and achieved early acceptance in the American medical system. Herbal medicine, on the contrary, is still left outside of the established medical field.

The relatively advanced institutionalization of acupuncture began with practitioner associations organized in the 1970s. Along the way, acupuncture schools were formed, resulting in 13 acupuncture and oriental medicine schools by 1981 [59]. State-level developments soon led to the birth of national organizations and orchestrated examining bodies [60,61]. By 1982, a decade after the initial organizing efforts, acupuncture established itself as a self-sufficient medical profession with its own education, certification, and practitioner associations at the national level. Most state governments subsequently began legalizing the practice of acupuncture.

American acupuncture has also been established in other institutional measures. In 1996, the Food and Drug Agency (FDA) reclassified acupuncture needles as “safe and effective medical devices”, replacing the 1973 classification as experimental “investigational devices” [61]. The National Institute of Health (NIH) produced a consensus report in 1997 supporting the potential efficacy of acupuncture [39]. Over 65 schools and colleges confer either a 3-year Master of Acupuncture degree, a 4-year Master of Acupuncture and Oriental Medicine degree, or a Doctor of Acupuncture and Oriental Medicine [62]. There are over 27,000 licensed acupuncturists in 45 states, five of which even allow acupuncturists to hold the title of primary care practitioner (PCP) who can perform biomedical test requests and patient referrals to other specialists. Insurance coverage is relatively limited yet. Federal healthcare schemes do not cover acupuncture and less than a quarter of state Medicaid programs cover acupuncture [63]. Eleven states have private insurance mandates [64]. About 47% of private policies cover acupuncture and they limit reimbursement to visits only to physician acupuncturists or pre-designated acupuncturists [65].

The current institutional status of herbal medicine is much less prominent than acupuncture. There is no sole medical degree of herbal medicine or certification of herbalists. Instead, acupuncture schools have gradually promoted herbal medicine by expanding the scope of practice by acupuncturists. It is, however, still illegal for acupuncturists to practice herbal medicine in half of the states [62]. Chiropractors, naturopaths, and midwives may only occasionally practice herbal medicine [64]. A majority of herbalists are food and nutrition specialists and not medical practitioners. This institutional under-development is surprising, considering herbal medicine’s central position within Oriental medicine and its long tradition in Native American medicine ([66]: 190, [67]).

The current status of herbal medicine practiced mostly in food stores and not by medical professionals is related to the American history of food and drug regulation. Manufacturers of herbal medicine have managed to define herbal medicine as “dietary supplements”, a regulatory category for a subgroup of food under the 1994 Dietary Supplement Health and Education Act (DSHEA). Accordingly, herbal medicine is not required to be registered and approved by the FDA for safety or efficacy. Manufacturers are required only to report its post-marketing adverse side effects while herbs cannot be marketed to have any medical effects [68].

Competing efforts still exist to place herbal medicine under the category of legitimate medicine rather than dietary supplements. On one hand, medical doctors have requested to officially recognize the high physiological potency of herbal medicine and bring it under strict FDA regulations equivalent to drugs [69,70]. On the other, acupuncturists have tried to enlist herbal medicine under the scope of practice by acupuncturists. Meanwhile, manufacturers can supply herbal medicine directly to users without any mediation from physicians, nurses, acupuncturists, or herbalists.

### Results from the meta-analysis of treatment outcomes

#### Treatment effectiveness

I found a cross-national difference in the treatment effectiveness of acupuncture and herbal medicine in both CENTRAL and MEDLINE. Acupuncture was found to be more effective in Japan than in the U.S. Eighty four out of 108 Japanese trials and 87 out of 138 American trials in CENTRAL found it effective (78% vs. 63%; p-value for Pearson’s χ^2^ test of independence = 0.013). Eleven out of 115 Japanese reports and 40 out of 601 American reports in MEDLINE found it effective (10% vs. 7%; p-value = 0.266).

Correspondingly, acupuncture and herbal medicine were practiced differently in Japan and the U.S. First, acupuncturists had different skill levels. Experienced acupuncturists applied acupuncture needles in the following first trial, whereas, in the second trial, staff nurses and research assistants practiced acupuncture with minimal training.

“[State-licensed] senior acupuncturists (5 + years in practice) decided where to needle and junior acupuncturists (2 - years in practice) practiced the interventions”. (CN-00735199)

“Dr. Ji-Sheng Han … provided an in-depth training to Dr. Meade and Ms. Eldridge. They, in turn, trained study staff (research assistants and nurses)… This staff was certified to administer treatments”. (CN-00728884)

As regards herbal medicine, in explaining the puzzle that a Japanese *kampo* formula (i.e. *keishibukuryogan*), which had been found efficacious for menopausal symptoms in Japan, produced unexpected adverse effects (i.e. diarrhea) in 20% of the trial subjects in the U.S. and only marginal treatment effects, a report pointed out the deficient knowledge about the proper dosage of *kampo* for American women (CN-00810843). The American trial had simply followed the dosage instruction made in the Japanese context. Two more studies evidenced that cross-national outcome differences were related to more or less informed ways of practicing herbal medicine (CN-00482723; CN-00750876).

Second, American trials applied acupuncture in a fragmented and less effective way, compared to the holistic Japanese practices. For example,

“Acupuncture treatments were performed at the Harvard-Thorndike General Clinical Research Center at Beth Israel Deaconess Medical Center. … However, massage, herbal prescriptions (or any other medical prescription), moxa, cupping, and lifestyle modifying therapies were not allowed”. (CN-00610755)

“Limitation: A prescription of acupuncture at fixed points may differ from acupuncture administered in clinical settings, in which therapy is individualized and often combined with herbal supplementation and other adjunctive measures”. (CN-00511534)

Better outcomes were indeed found in a “whole system clinical trial” that adhered to the holistic practices (CN-00640342). A growing emphasis is placed on “pragmatic” trials designed to capture the overall impact of acupuncture as a whole system vis-à-vis the fragmented “drug model” of trials [71].

Third, treatment effectiveness was dependent upon whether acupuncture was coordinated with other conventional treatments. For example, two clinical trials conducted on the same site, on the same subjects (i.e. cocaine-dependent patients at the Yale School of Medicine), and with the same acupuncture treatment, produced different treatment results (CN-00403270). Acupuncture was ineffective in treating cocaine addiction in one trial; it was effective in the other. In the effective trial, subjects were additionally provided with a manual-based conventional psychological therapy called “coping skill therapy (CST)” which was absent in the ineffective trial. So, the study concluded that:

“acupuncture, a nonverbal treatment that does not in and of itself teach skills requisite for abstinence, may need to be embedded within an appropriate psychological framework in order to be effectively utilized. … The absence of the CST may have constituted a significant omission”. (CN-00403270)

#### Biomedicine–CAM synergy

In the Pearson χ^2^ test of independence among the CENTRAL trials, herbal medicine was found to produce synergy with biomedicine more often in Japan than in the U.S. (13% vs. 3%; p-value < 0.001). The cross-national difference was less obvious in acupuncture (22% vs. 11%; p-value = 0.028). In Japan, herbal medicine was found to be as synergic with biomedicine as acupuncture was (13% vs. 11%; p-value = 0.674). In the U.S., to the contrary, herbal medicine was significantly less synergic than acupuncture (9% vs. 30%; p-value < 0.001).

The details of biomedicine–CAM synergy were identifiable in two categories. First, synergy was represented in an additive manner. For many medical conditions, the addition of acupuncture or herbal medicine to biomedical treatments often produced effective treatment results, compared to the ineffective lone treatment of either biomedicine or CAM. The addition sometimes produced greater effects, compared to the effects of either lone treatment.

For example, acupuncture produced additive effects on infertile women under in-vitro fertilization treatments (CN-00730574; CN-00768745; CN-00700432), opioid drug addicts on medications (CN-00728884), cancer survivors under biomedical treatments for neck pain (CN-00761076), people on medications for chronic musculoskeletal pains (CN-00720285; CN-00457236), and people on medications for irritable bowel syndrome (CN-00698848). Acupuncture also produced analgesic or anesthetic benefits to pediatric surgery (CN-00734541), cardiac surgeries (CN-00720700), oncological surgeries (CN-00579120; CN-00664750), and dental surgeries (CN-00330168).

Herbal medicine produced additive synergy among people with rheumatoid arthritis on a conventional medication (CN-00729697), surgical patients under a variety of post-operative cares for surgery-induced intestinal bowel syndrome, inflammation, liver dysfunction, and pains (CN-00668598; CN-00686725; CN-00410068; CN-00556925; CN-00347524; CN-00434525; CN-00609214; CN-00512697; CN-00373691; CN-00793124; CN-00790633; CN-00793458), and cancer patients under radiation therapy (CN-00132749; CN-00347726; CN-00330625).

Second, biomedicine–CAM synergy was revealed in an interactive and corrective way, as well. In this case, CAM was used to relieve the adverse side effects of biomedical treatments and help patients to continue biomedical treatment until they got desirable treatment outcomes from biomedicine.

For example, acupuncture helped HIV patients to continue HAART (Highly Active Anti-Retroviral Therapy) by alleviating gastrointestinal syndrome in HIV patients on HAART, such as diarrhea, nausea, and vomiting (CN-00558827; PMID 21705396). It also reduced vasomotor symptoms, such as hot flashes and night sweats, developing from anti-estrogen hormone therapy on breast cancer patients and, thus, helped the patients to continue biomedical cancer treatments (CN-00700744; CN-00733070). At the same time, acupuncture produced fewer adverse effects than venlafaxine (a conventional antidepressant for cancer patients on anti-estrogen treatment) and more positive outcomes, such as energy and sex drive (CN-00733070). In addition, acupuncture was found effective for managing a common side effect (joint pains) of the AI hormone therapy for early breast cancer patients who often discontinued the hormone therapy due to the side effect (CN-00649930; CN-00729624). Acupuncture also slowed the decrease in the ability of bone marrow to produce blood cells, a side effect of chemotherapy for gynecologic cancers (CN-00722277).

In herbal medicine, the *kampo* remedies *maoto* and *shosaikoto* were found effective in reducing discomfort, general malaise, and arthralgia induced by the established interferon treatment for patients with chronic hepatitis C, while not reducing the interferon’s antiviral effects and sometimes improving its effects (CN-00794206; CN-00793700; CN-00472711). Another group of herbal remedies helped cancer patients to continue biomedical treatments, by relieving the side effects of cancer treatments, such as neurotoxicity, diarrhea, and insomnia (CN-00728144; CN-00437238; CN-00779065). Examples in other contexts were *juzentaihoto* for addressing anemia in patients on hemodialysis (CN-00667345), *rikkunshi-to* for adverse gastrointestinal symptoms among people on anti-depressant fluvoxamine (CN-00795653), and maca root for anti-depressant SSRI-induced sexual dysfunction (CN-00665598).

#### Adverse treatment outcomes of CAM

Among the CENTRAL trials, I found no reports of adverse treatment outcomes from acupuncture in Japan or the U.S., whereas there were several trials of herbal medicine in the U.S. where unforeseen difficulties developed. Reported events were diarrhea caused by a Japanese *kampo* formula *keishibukuryogan* used for menopausal symptoms (CN-00810843), high blood pressure among users of athletic performance-enhancing dietary supplements (CN-00647862), increased blood pressure and heart rates among people who took bitter orange for controlling obesity (CN-00553213), and the damaging effects on male reproductive cells from St. John’s wort, ginkgo, and echinacea in high concentrations (PMID 10065791).

In the MEDLINE reports as well, herbal medicine was found to produce adverse outcomes more often than acupuncture. Adverse treatment outcomes were mentioned in 23% of the 3,485 herbal medicine reports vs. 5% of the 716 acupuncture reports (p-value < 0.001). In the U.S., this contrast between herbal medicine and acupuncture was greater (25% vs. 4%; p-value < 0.001), whereas the contrast was significantly weaker in Japan (16% vs. 10%; p-value = 0.078). Put in a cross-national perspective, herbal medicine produced more adverse outcomes in the U.S. than in Japan (25% vs. 17%; p-value < 0.001); acupuncture produced adverse outcomes less often in the U.S. than in Japan (4% vs. 10%; p-value = 0.010).

Content analysis revealed some of the specific occasions that contributed to producing these adverse events. Out of the 12 Japanese reports of adverse effects from acupuncture, a majority reported minor injuries, such as localized argyria and minor hemorrhages, caused by acupuncture needles left in the body (PMID 1464937; PMID 11444889; PMID 12459543; PMID 20934166). In most cases, tiny acupuncture needles were intentionally and permanently embedded in patient bodies for therapeutic purposes according to Japanese acupuncture theory. On the other hand, major injuries were often caused by accidentally broken needles during laypeople’s self-treatments.

Another common category of the adverse effects was virus infection through improperly sterilized acupuncture needles, such as hepatitis C virus infections (PMID 9816817; PMID 8194707; PMID 7689501; PMID 1908912) and other infections (PMID 1488961). Unlike the reports of minor injuries, these infection reports originated in earlier years. According to more recent reports, the association between acupuncture and infection was found to be weak in hepatitis C virus (PMID 7681088; PMID 8329759; PMID 9033214; PMID 9094854; PMID 10756668) and HIV (PMID 8329759; PMID 9094854).

Reports of needle-induced infections existed in the U.S. as well, such as the contraction of hepatitis C (PMID 22239506; PMID 16379222; PMID 10235216) and hepatitis B (PMID 3341362; PMID 3944549; PMID 3417241). Some other U.S. reports were about subarachnoid hemorrhages and spinal injuries in a Latino immigrant who visited an unproven thoracic acupuncturist in California (PMID 1464937), forgotten needles and minor hemorrhages from poorly-trained practitioners (PMID 11829162; PMID 11874310), minor skin problems from moxibustion (PMID 3965232), contact dermatitis from needles, and unspecific general worries about acupuncture (PMID 1137233; PMID 4406704; PMID 4408150; PMID 4607022; PMID 4590887). Across these reports, the lack of practitioner skills or institutional regulations on practitioner education was found to be a cause of the adverse events.

#### Negative biomedicine–CAM interactions

Out of the 3,485 MEDLINE reports on herbal medicine, only a small fraction (39 reports; 1.1%) reported negative interactions with biomedical drugs. While reports of negative interactions were rare in general, I found a significant cross-national difference in the proportion of these reports among all the reports on herbal medicine (0.1% in Japan vs. 1.6% in the U.S.; p-value < 0.001). Thirty eight out of the total 39 reports of negative interactions were from the U.S., except for one Japanese report about a 48 year old woman who developed extensive red round rashes over her legs hours after she took *kakkoto* for general fatigue. This woman was already taking several medications for atypical psychosis when she took *kakkoto*. These drugs in combination with the herbal medicine *kakkoto* reportedly developed the adverse interactions (PMID 14674921).

A majority of U.S. reports (24 out of 38) were about popular herbal supplements, such as garlic, ginkgo, echinacea, ginseng, St John’s wort, and kava, and their potential interactions with drugs among the general population as well as among patients with cancers, cardiovascular conditions, or endocrine disorders. These reports stressed mostly that adverse interactions could be prevented by doctors and nurses who elicit the history of patients’ herbal medicine use (PMID 10737289; PMID 19390395) and prescribe herbal supplements appropriately based on this history (PMID 15581442). The following is an exemplar quote about how drug–herb interactions could complicate biomedical treatments.

“CAM and antiretroviral therapies [for HIV patients] can produce side effects, such as weight loss. Additional drug interactions with nutritional supplements can increase health complications. … Providers may attribute these adverse effects to the antiretroviral drug, prompting them to incorrectly switch [antiretroviral] medications. This can be expensive and may lead to increased antiretroviral resistance. These and similar situations can be avoided by creating and maintaining an open dialogue about CAM use”. (PMID 19181175)

Among the CENTRAL trials, there was no report of acupuncture producing adverse treatment interactions with biomedicine. On the other hand, among trials of herbal medicine, I found three types of adverse treatment interactions between herbal medicine and biomedicine. First, herbal medicine interfered with biomedical treatments as follows: St. John’s wort’s interference with contraceptives (CN-00528293), oncological drugs (CN-00491970), and intestinal P-glycoprotein (CN-00470658); American ginseng’s interference with Warfarin’s anti-coagulant mechanism in postoperative care (CN-00467683). Second, herbal medicine inadvertently concentrated the effects of biomedical drugs. For example, ephedrine’s co-utilization with the caffeine in herbal supplements (e.g. green tea) for obesity inadvertently produced abnormal cardiovascular, metabolic, and hormonal responses (CN-00470658). Third, it caused a delay in seeking relevant medical treatments, such as the delay of professional biomedical treatments for asthma during patients’ self-care with herbal supplements (PMID 9438488). Children were significantly less likely to receive vaccinations when they were seeing a naturopathic physician (PMID 19760163). There were negative associations between CAM use and Chlamydia screening and between naturopathy and mammography among women (PMID 19630554).

There were several specific treatment contexts in which drug–herb interactions occurred most often. First, drug–herb interactions occurred in “cross-cultural” or “multi-cultural” settings in the U.S. Border cities between the U.S. and Mexico (e.g. El Paso, Texas) were examined in a number of studies as a difficult social context where the risk of drug–herb interactions was higher than that of the general U.S. population (PMID 16396061; PMID 19552494; PMID 18928136). Studies pointed to two contributing factors: 1) higher utilization rates of herbal medicine among residents in border cities and 2) herbal medicine users’ cross-border healthcare utilization that made it difficult for healthcare providers on both sides to trace and coordinate patients’ plural medical behavior.

Another group of reports revealed that adverse drug–herb interactions were more probable among U.S. immigrant minorities, such as Mexican-Americans (PMID 17405676) and Slavic-Americans (PMID 17900071). In addition, a survey of six university-affiliated outpatient clinics in California (PMID 17405676) reported that 85 adverse drug–herb interactions were found in 49 patients (40% of herbal medicine users); 12 potential adverse drug–herb interactions were found in 8 patients (7% of the users); among these 12 cases, 8 cases were hypoglycemia among diabetics who took prickly pear cactus along with biomedical drugs. These 8 cases were all from first-generation Mexican-Americans who also tended to use herbal medicine more often than others.

In addition, several categories of users were identified as most likely to experience adverse treatment interactions. Many cancer patients were using herbal medicine concurrently with anti-cancer treatments. However, a significant number of these users did not consult their doctors (PMID 15856334; PMID 14991387). The same situation existed among elderly people who were highly subject to “polypharmacy” (i.e. multiple drugs) and “polyherbacy” (i.e. multiple herbal remedies) for high comorbidity (PMID 17785609; PMID 15018694; PMID 15037491). Another category was illicit stimulant users among whom certain herbal remedies (e.g. yohimbe), that have high potentials to interact adversely with biomedical drugs for treating the stimulant users, were wrongfully promoted as sexual enhancers (PMID 18570167).

## Discussion

Japan has integrated acupuncture and herbal medicine more systemically and formally into the national medical system than the U.S. In addition, herbal medicine has been more closely integrated with biomedicine than acupuncture in Japan. In the U.S., to the contrary, acupuncture has been incorporated in the system whereas herbal medicine has not. Although institutionalized more discursively than systemically, American acupuncture has been given a similar status in the American medical system as Japanese acupuncture has in the Japanese system. American acupuncture even seems to be positioned better. Whereas Japanese acupuncture has been detached from herbal medicine and the whole system of Japanese traditional medicine, American acupuncture has not been explicitly detached from the whole Oriental medical system. Put in an order, Japanese herbal medicine has been integrated into the national medical system the most and American herbal medicine the least; American acupuncture and Japanese acupuncture have fallen in the middle.

The treatment outcomes of acupuncture and herbal medicine in Japan and the U.S. correspond with these differences in institutionalization. First, acupuncture and herbal medicine that were carefully coordinated with other treatment options in CENTRAL clinical trials produced better outcomes, such as more occasions of desirable outcomes and biomedicine–CAM synergies with no reports of adverse treatment effects, compared to MEDLINE reports that included uncontrolled and uncoordinated real-world treatment cases. Second, among CENTRAL trials and among MEDLINE reports, treatment outcomes were the most favorable for Japanese herbal medicine and the least favorable for American herbal medicine. The outcomes of American acupuncture and Japanese acupuncture fell in the middle. Thus, in Japan, herbal medicine was found to be more effective than acupuncture; in the U.S., acupuncture was found more effective than herbal medicine.

This correspondence between the institutionalization of acupuncture and herbal medicine on one hand and the CAM treatment outcomes on the other provides empirical evidence to the notion that the co-utilization of CAM along with biomedicine involves difficulties for users and practitioners due to theoretical and political tensions between the two [16,32,72]. In addition, my paper further enriches this notion by adding that these difficulties can be relieved and result in various treatment outcomes of CAM, depending on the extent to which CAM interventions are coordinated with biomedicine. Moreover, through content analysis, my paper provides an in-depth understanding of social contexts in which the co-utilization of CAM and biomedicine produces negative biomedicine–CAM interactions and adverse CAM treatment outcomes. These contexts existed at several levels, such as regulatory policies, practitioner expertise, user–provider communication, and users’ cross-cultural health behavior. At the same time, various types of synergic benefits were evident when CAM and biomedicine were coordinated in institutional settings.

This paper also supports with ample evidence the ethnographic insight that institutional settings affect how unconventional CAM interventions are perceived and practiced [29]. It further advances this insight by additionally finding that it is not only the perception and expectation of medical practices but also their concrete treatment outcomes that are affected by institutional settings. It is possible that effective medical interventions are more likely to be institutionalized, which is another empirical question that has yet to be answered. Meanwhile, based on empirical episodes in medical reports, my current evidence demonstrates that interventions of a similar nature lead to varying outcomes in different institutional settings. When the nature (e.g. CAM) is doubted by dominant theories (e.g. biomedicine) and politics in the medical field, the sociological perspective that I practiced in this paper seems to be the one that the sociology of the effects of medicine and science needs to carry forward. The literature already evidences that unorthodox and alternative entities are being incorporated into medicine and science in various manners [73]. It would be even more fruitful to investigate what effects these different manners of incorporation produce.

In the sociology of the effects of medicine, broader social contexts of medical practices have often been held accountable for the varying treatment outcomes of biomedical interventions. This wisdom, however, has not been seriously applied when considering the varying outcomes of popular CAM, such as acupuncture and herbal medicine. The varying outcomes of CAM have instead been dominantly ascribed to their arguably inferior and incomplete nature as medical technologies. This asymmetry is uneasy. When an intervention in biomedicine does not deliver its expected outcomes, studies have implied that the relationship between medicine and social contexts should be addressed. In CAM, on the other hand, the dominant view is that medicine by itself has to be reengineered, corrected, and improved technologically. Without addressing this asymmetry, the sociology of medicine runs the risk of becoming one of the “engines” [74] of “biomedicalization” [75], by inadvertently stressing social contextual re-organization tuned only to biomedicine and, simultaneously, the biomedical transformation of CAM. This paper provides a counter-perspective. It is social contexts as well as medicine by itself that generate varying treatment outcomes in CAM. This paper suggests that social contexts also need to be tuned to CAM in order to produce better treatment outcomes.

Methodologically, this paper’s content analysis can be labeled as a sociological meta-analysis, which is in distinction to meta-analysis in the medical science community. Medical meta-analysis aims to produce a model of the universally consistent effects of medicine by bracketing the contextual differences out of consideration. My sociological meta-analysis, to the very contrary, purports to generate a model of the differential effects of medicine by shedding light on the very contextual differences. There is still room for improvement on this front. My current meta-analysis is largely a bivariate analysis of the relationship between treatment outcomes and social contexts (i.e. the U.S. versus Japan) both quantitatively and qualitatively. It needs to be expanded into a multi-variate analysis that can verify the robustness of the findings even in the presence of other covariates. On the qualitative front, the characteristics of social contexts can be specified further.

## Conclusion

Acupuncture and herbal medicine, which have tensions with the mainstream biomedicine, are institutionalized differently in Japan and the U.S. Depending on the extent to which these CAM interventions are coordinated with biomedicine in the national medical system, they produce different treatment outcomes. When coordinated with biomedicine, acupuncture and herbal medicine produce more beneficial treatment outcomes.

## Appendix

One can get the MEDLINE papers that I used for content analysis, by copying the following search queries into the search box of PubMed (i.e. the user interface for the MEDLINE database). There could be minor discrepancies between the materials in this paper (retrieved on May 9, 2012) and the materials that one gets currently from the interface. These discrepancies are all additional materials that are deposited to MEDLINE since I accessed it.

A. the subset of CAM: cam[sb]

B. acupuncture reports from Japan: japan[MeSH Terms] AND (acupuncture[MeSH Terms] OR acup* OR acupoint[MeSH Terms] OR ear acupuncture[MeSH Terms] OR auricular acupuncture[MeSH Terms] OR analgesia, acupuncture[MeSH Terms] OR anesthesia, acupuncture[MeSH Terms])

C. acupuncture reports from the U.S.: united states[MeSH Terms] AND (acupuncture[MeSH Terms] OR acup* OR acupoint[MeSH Terms] OR ear acupuncture[MeSH Terms] OR auricular acupuncture[MeSH Terms] OR analgesia, acupuncture[MeSH Terms] OR anesthesia, acupuncture[MeSH Terms])

D. herbal medicine reports from Japan: japan[MeSH Terms] AND (“*herb*” OR “plant extract*” OR “kampo” OR “kanpo” OR herbal medicine[MeSH Terms] OR herbal preparation[MeSH Terms] OR herb therapy[MeSH Terms] OR herbal therapy[MeSH Terms] OR herb, medicinal[MeSH Terms] OR drug herb interaction[MeSH Terms] OR herb drug interaction[MeSH Terms] OR medicinal plant[MeSH Terms] OR medicinal herb[MeSH Terms] OR phytotherapy[MeSH Terms])

E. herbal medicine reports from the U.S.: united states[MeSH Terms] AND (“*herb*” OR “plant extract*” OR “kampo” OR “kanpo” OR herbal medicine[MeSH Terms] OR herbal preparation[MeSH Terms] OR herb therapy[MeSH Terms] OR herbal therapy[MeSH Terms] OR herb, medicinal[MeSH Terms] OR drug herb interaction[MeSH Terms] OR herb drug interaction[MeSH Terms] OR medicinal plant[MeSH Terms] OR medicinal herb[MeSH Terms] OR phytotherapy[MeSH Terms])
